# Inhibitory Effect of the Hexane Fraction of the Ethanolic Extract of the Fruits of *Pterodon pubescens* Benth in Acute and Chronic Inflammation

**DOI:** 10.1155/2013/272795

**Published:** 2013-07-21

**Authors:** Jaqueline Hoscheid, Ciomar Aparecida Bersani-Amado, Bruno Ambrósio da Rocha, Priscila Miyuki Outuki, Maria Angélica Raffaini Cóvas Pereira da Silva, Diego Lacir Froehlich, Mara Lane Carvalho Cardoso

**Affiliations:** ^1^Universidade Estadual de Maringá, Centro de Ciências da Saúde, Departamento de Farmácia, Avenida Colombo, 5790 bloco K80. Zona Sete, 87020-900 Maringá, PR, Brazil; ^2^Universidade Estadual de Maringá, Centro de Ciências da Saúde, Departamento de Farmacologia e Terapêutica, Avenida Colombo, 5790 bloco K68. Zona Sete, 87020-900 Maringá, PR, Brazil; ^3^Pontifícia Universidade Católica do Paraná-PUCPR, Escola de Ciências Agrárias, Avenida da União, 500. Jardim Coopagro, 85902-532 Toledo, PR, Brazil

## Abstract

Fruits of *Pterodon pubescens* Benth have been used traditionally for the treatment of rheumatism, sore throat, and respiratory disorders, and also as anti-inflammatory, analgesic, depurative, tonic, and hypoglycemic agent. The study was aimed at evaluating the anti-inflammatory activity of the hexane fraction of an ethanolic extract of *P. pubescens* fruits. The oil from *P. pubescens* fruits was extracted with ethanol and partitioned with hexane. The anti-inflammatory activity was measured with increasing doses of the hexane fraction (FHPp) by using a carrageenan-induced rat model of pleurisy and a rat model of complete Freund's adjuvant-induced arthritis by using an FHPp dose of 250 mg/kg for 21 days. Treatment with an FHPp resulted in anti-inflammatory activity in both models. The results of biochemical, hematological, and histological analyses indicated a significant decrease in glucose, cholesterol, and triglycerides levels (18.32%, 34.20%, and 41.70%, resp.) and reduction in the numbers of total leukocytes and mononuclear cells. The FHPp dose of 1000 mg/kg induced no changes in behavioral parameters, and no animal died. The results of this study extend the findings of previous reports that have shown that administration of extracts and fractions obtained from species of the genus *Pterodon* exhibits anti-inflammatory activity and lacks toxicity.

## 1. Introduction

*Pterodon pubescens *Benth (Leguminosae), commonly known as “faveira,” “sucupira,” or “sucupira branca,” is a tree native to Brazil and is distributed across its central region [1]. The *P. pubescens* fruit is traditionally used in ethnomedicine as an infusion [2], in small doses and at regular time intervals as an anti-inflammatory, analgesic, tonic, depurative [3, 4], and hypoglycemic agent [5]. 

Active metabolites from species belonging to the genus *Pterodon* are being isolated and their medicinal properties are being investigated. Several studies have shown that oil from the fruit of *P. pubescens* has a significant level of furanoditerpenes, which are directly involved in the pharmacological activities of this fruit [6–9]. The hydroalcoholic extract of the *P. pubescens* fruit when administered orally (gavage) shows antinociceptive properties [10, 11] and anti-inflammatory activity in a mouse model of collagen-induced arthritis [12, 13] without altering hematological, clinical, biochemical, and histopathological parameters [12]. The ethanol extract of the fruit also demonstrates anti-inflammatory activity [7] and suppressive effects on the immune response mediated by T and B lymphocytes [2]. The goal of our current study was to evaluate the anti-inflammatory activity of the hexane fraction (FHPp) of the ethanol extract obtained from the fruit of *P. pubescens* by using a rat model of pleurisy and a rat model of arthritis induced by complete Freund's adjuvant (CFA).

## 2. Material and Methods

### 2.1. Animals

The experimental procedures were approved by the Ethics Committee of the Universidade Estadual de Maringa (protocol number 018/2011). The carrageenan induced pleurisy model was established using male Wistar rats (weight: 200–220 g), and the adjuvant-induced arthritis model was established using male Holtzman rats (weight: 170–200 g); male Swiss mice (weight: 20–30 g) were used for a toxicity study. The animals were housed at 22 ± 2°C under a 12 h light/12 h dark cycle with free access to food and water.

### 2.2. Collection of the Vegetal Material and Extractive Process

*P. pubescens *Benth fruits were collected from Nossa Senhora do Livramento, M.T., Brazil (15°89′ S; 56°41′ W) in May 2010 and identified by Dr. Germano Guarim Neto from the Herbarium of Federal University of Mato Grosso, and the voucher specimen was deposited in the Herbarium of Maringá State University, under number 20502. The dried fruits (30 g) were extracted with ethanol 99.5% P.A. (600 mL) by turbo extraction and then filtered. The filtrate was partitioned with water:hexane (1 : 1). The organic solvent was evaporated in a vacuum evaporator to yield the hexane fraction (FHPp) as previously described [14].

### 2.3. Acute Carrageenan-Induced Inflammatory Reaction in the Pleural Cavity of Rats

The test was conducted according to the method described by Vinegar et al. [15]. Groups of rats (*n* = 5 per group) were pretreated by oral gavage with a solution of FHPp (125, 250, and 500 mg/kg) in 2% Tween 80 in water, dexamethasone (0.5 mg/kg) as a standard drug, or a solution of 2% Tween 80 in water as a control. After 1 h, all animals received an intrapleural injection of carrageenan (200 *μ*g/animal). Four hours later, the animals were anesthetized with xylazine (10 mg/kg), the pleural exudate was collected, and its volume was determined. The number of leukocytes that migrated to the exudate was measured using a Neubauer chamber cell counting.

### 2.4. Induction and Evaluation of Experimental Arthritis

Adjuvant-induced arthritis (AIA) was produced on day 0 by an intradermal injection of 100 *μ*L of a CFA suspension (heat-inactivated *M. tuberculosis* suspended in mineral oil at a concentration of 0.5% w/v) into the left hind paw [16]. The development of AIA was assessed by paw volume changes and appearance of secondary lesions. The volume of the injected and noninjected hind paw was determined by digital plethysmography. The results were expressed as the increase in paw volume relative to the initial volume. Measurements were performed over a 21-day period. The severity of secondary lesions was evaluated using a numerical grading system [17]. Briefly, points were assigned to each of the following events: appearance of nodules in the tail (+1); appearance of nodules in one or both ears (+1 or +2); and appearance of swelling in one or both forelimbs (+1 or +2). The severity of the secondary lesions was graded from 0 to 5, with 0 indicating the absence of lesions. Body weight was assessed daily at 15 h.

In this experiment, the animals (*n* = 5 per group) were divided into 3 treatment groups: (1) animals with arthritis which received only distilled water (AIA control); (2) animals with arthritis which received Tween 80 solution at 2% in distilled water (AIA + Tween); and (3) animals with arthritis which received 250 mg/kg of FHPp suspended in 2% Tween 80 (AIA + FHPp). Treatment was performed daily by oral administration (gavage) for a period of 21 days starting on the day of the intradermal adjuvant injection.

### 2.5. Subacute Toxicological Evaluation of FHPp

#### 2.5.1. Hematological Examination

An aliquot of blood from the distal end of the rats' tails was collected for the determination of total white blood cell count and white blood cell differentials prior to induction of AIA (day 0) and after 21 days of treatment.

#### 2.5.2. Biochemical Examination

The biochemical analyses for determination of glucose, cholesterol, triglycerides, urea, creatinine, alanine aminotransferase (ALT), and aspartate aminotransferase (AST) levels were performed using blood samples collected with heparin from the abdominal vena cava on treatment day 21. The blood was centrifuged and stored at −4°C until use. The analyses were performed with a spectrophotometer (Bio-BIOPLUS 2000) using biochemical kits (analyzed).

#### 2.5.3. Organ Weights and Histopathological Examination

Animals were killed 21 days after AIA. During necropsy, the weight of several organs (the liver, kidney, spleen, thymus, adrenal gland, and lymph nodes) was determined and expressed as weight (*g*) of fresh organ/100 g animal body weight.

The livers and kidneys were preserved in a formalin solution for routine histological processing. Then, these were embedded in paraffin, sectioned, and stained with hematoxylin and eosin for histopathological examination according to conventional histological methods.

### 2.6. Acute Toxicity

For evaluation of toxicity, Swiss mice (*n* = 6) were fasted for 15 h. Each animal was orally administered 1000 mg/kg of the FHPp suspended in 2% Tween 80. During the first 4 h after administration, behavioral parameters (motor activity, convulsions, piloerection, salivation, and sedation) were observed and described according to Malone and Robichaud [18]. Body weight was assessed for a period of 7 days.

### 2.7. Evaluation of Food Intake and Body Weight of Rats Treated with FHPp

After induction of arthritis by CFA, rats in the treatment group received a solution of 250 mg/kg FHPp in 2% Tween 80 and distilled water, while control rats received only distilled water. All rats were kept in metabolism cages for 21 days to assess food and water intake and to collect urine. Body weights were evaluated daily. 

### 2.8. Statistical Analysis

Data are presented as means ± standard error of the mean (S.E.M.). Results were statistically analyzed using GraphPad software (GraphPad Software, Inc., San Diego, CA, USA). Student's *t*-tests for unpaired data (two-tailed) or one-way analysis of variance (ANOVA) followed by post hoc Tukey tests was performed. *P* values less than 0.05 were considered statistically significant.

## 3. Results

### 3.1. Effects of FHPp in the Rat Model of Carrageenan-Induced Pleurisy

In animals treated with vehicle (control group), an intrapleural injection of carrageenan caused accumulation of a pleural exudate and intense recruitment of leukocytes to the inflamed site. Treatment of rats with FHPp at doses of 250 and 500 mg/kg significantly reduced the volume of the inflammatory exudate by 28.7% and 26.6%, respectively. However, only the higher dose (500 mg/kg) caused a significant decrease in the number of leukocytes (Table 1). As expected, animals treated with dexamethasone (0.5 mg/kg) showed no inflammatory cell infiltration.

### 3.2. Complete Freund's Adjuvant-Induced Arthritis Model

#### 3.2.1. FHPp Effects in the AIA Model

**Figure 1:**
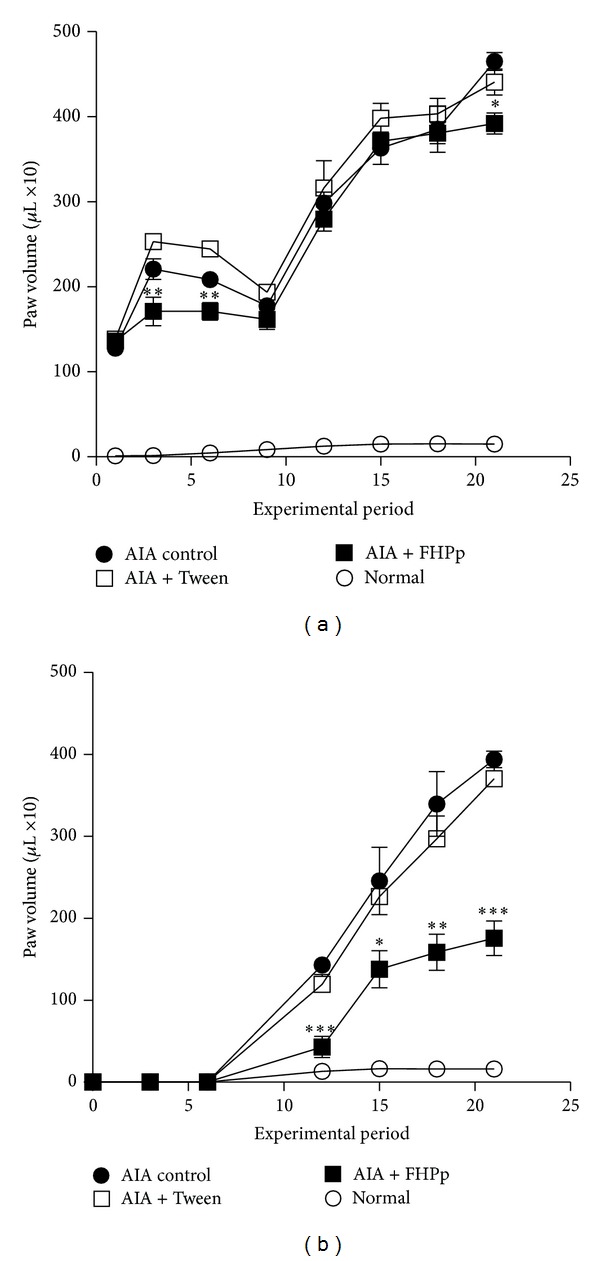
Development of the inflammatory response in the hind paws of rats: (a) left paw injected with complete Freund's adjuvant and (b) right paw, not injected. Each point represents the mean ± S.E.M. (*n* = 5). *P < 0.05;  **P < 0.01;  ***P < 0.001 compared to the AIA control group. One-way ANOVA post hoc Tukey test.

We observed an intense inflammatory reaction in the left hind paw following injection of complete Freund's adjuvant (CFA) on the first day, and the reaction progressively worsened during the following 21 days. An inflammatory response in the noninjected paw (right hind paw) was observed from the tenth day after the induction of AIA and increased gradually until day 21. Treatment of rats with FHPp at a dose of 250 mg/kg did not affect the development of edema in the paw injected with CFA but significantly reduced the edema in the right foot, which was not injected with CFA. Treatment of rats with Tween (used with vehicle) did not affect the inflammatory response in either paw as compared to the control group of AIA rats. The results are shown in Figures 1(a) and 1(b).

As shown in Table 2, treatment with FHPp delayed the onset of the secondary injury that developed starting on the tenth day after the induction of arthritis. In addition, secondary lesions developed less aggressively in the rats treated with FHPp.

#### 3.2.2. Hematological Examination

Compared to the blood obtained from the control rats, the blood obtained from rats after induction of AIA showed a significant increase in the number of total and differential leukocytes. However, daily treatment with FHPp at a dose of 250 mg/kg significantly reduced the total number of leukocytes and mononuclear cells compared to the corresponding numbers in the controls (Table 3).

#### 3.2.3. Biochemical Analysis

Statistically significant changes were observed in plasma levels of glucose, cholesterol, and triglycerides in AIA rats treated with FHPp compared to the AIA control rats and normal animals.

Compared to the untreated group with arthritis, FHPp-treated rats with arthritis did not show significantly altered levels of creatinine, urea, ALT, or AST. However, ALT and urea levels were significantly different in the 2 groups as compared to the corresponding levels observed in normal Holtzman rats (Table 4).

#### 3.2.4. Relative Weight of Organs and Histological Evaluation

**Figure 2:**
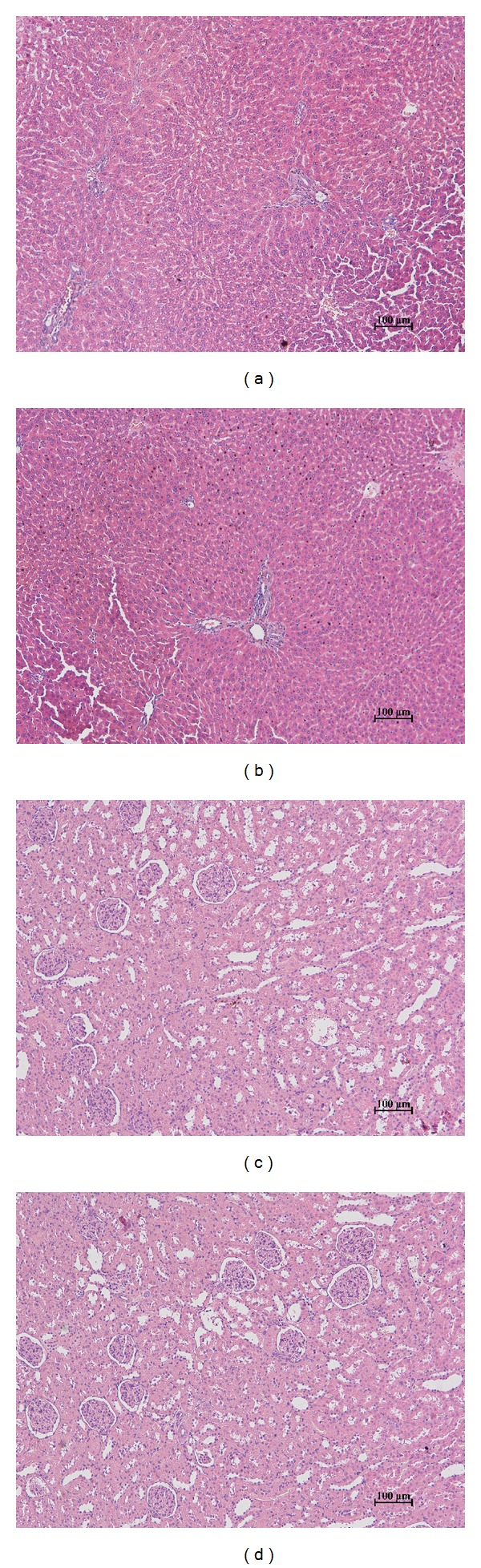
Livers (portal triad regions) of animals that received intraplantar injection of CFA, treated orally, 21 days, with (a) water or (b) FHPp 250 mg/kg and kidneys (renal glomeruli and tubules) of rats that received intraplantar injection of CFA, treated orally, 21 days, with (c) water or (d) FHPp 250 mg/kg.

After 21 days of FHPp treatment, there were no significant changes in the relative weight of lymphoid organs (Table 5). Histological analysis demonstrated the absence of lesions in kidney and liver samples from animals treated with FHPp (Figure 2).

#### 3.2.5. Evaluation of the Toxicity of FHPp

In acute toxicity studies (hippocratic test), treatment with FHPp at doses up to 1000 mg/kg caused no observable changes in behavioral parameters or body weight, and no animal died.

#### 3.2.6. Assessment of Food Intake and Body Weight

The weight of the animals increased progressively until the tenth day, when the systemic manifestations of the disease began, at which time a slight weight loss occurred.

**Figure 3:**
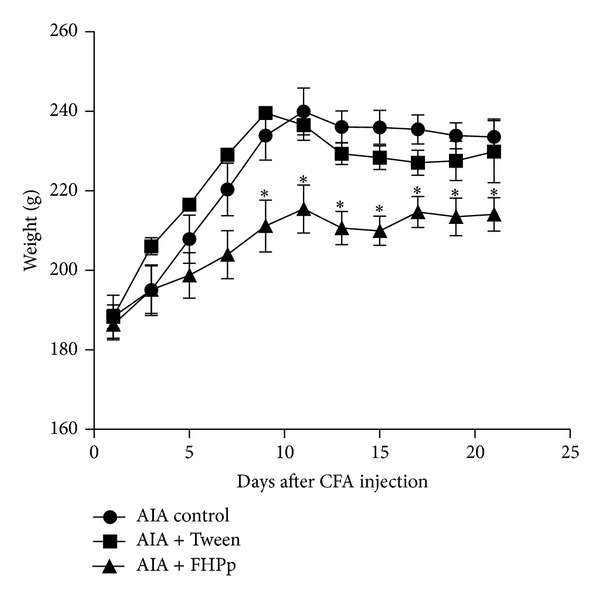
Effect of FHPp (250 mg/kg) on the weight of the arthritic animals. Each point represents the mean ± S.E.M. (*n* = 5).  *P < 0.05 compared to the AIA control group. One-way ANOVA post-hoc Tukey test.

There were no statistically significant changes in water or food intake or in the volume of urine produced between the groups AIA control and AIA + FHPp. However, the group treated daily with 250 mg/kg of FHPp showed significantly less weight gain than the other groups (AIA Control) (Figure 3).

## 4. Discussion

The present study evaluated the effects of the hexane fraction of the ethanolic extract of *P. pubescens* administered orally in two *in vivo* inflammatory models: carrageenan-induced inflammatory reaction in the pleural cavity and CFA-induced arthritis. The results showed that FHPp treatment induced anti-inflammatory effects in both models.

Our results are consistent with those of Carvalho et al. [6], who evaluated the acute anti-inflammatory activity of the hexane extract of *P. emarginatus* in a carrageenan-induced peritonitis model. They suggested that the activity of the extract might be related to inhibition of prostaglandin release and other mediators of the kinin system. Other studies have provided evidence that the ethanol extract of fruit from *P. pubescens* can suppress the humoral and cellular immune system by inhibiting the proliferation of lymphocytes and nitrite production by macrophages, acting as an immunomodulator with potential application in the treatment of patients with inflammatory or autoimmune diseases [2]. Silva et al. [7] demonstrated that the antiedematogenic activity of the alcoholic extract and fractions of *P. pubescens* was due to the compounds identified as geranylgeraniol and farnesol and a complex mixture of furanoditerpenes.

Several diterpenoids have been isolated from the fruits of *Pterodon* species. In a recent study, the diterpene 6*α*,7*β*-dihydroxy-vouacapan-17*β*-oic acid isolated from *P. emarginatus* and administered at a dose 50 mg/kg body weight significantly inhibited inflammation [19]. Thus, there is a high possibility of anti-inflammatory diterpenoids being present in the genus *Pterodon*. Earlier studies by our group have identified the presence of 3 diterpenes in FHPp that may be directly involved in the observed activity: methyl 6*α*-acetoxy-7*β*-hydroxyvouacapan-17*β*-oate, methyl 6*α*-hydroxy-7*β*-acetoxyvouacapan-17*β*-oate, and 14,15-epoxygeranylgeraniol [14].

Arthritis is a chronic, autoimmune inflammation of the joints that is especially exacerbated by the functions of macrophages and lymphocytes, which in turn contributes to the formation of paw edema. Sabino et al. [20] showed that the ethanol extract of *P. pubescens* at doses of up to 8 g/kg could inhibit lymphocyte proliferation and expression of proinflammatory cytokines accompanied by the induction of apoptosis, which consequently caused less leukocyte migration compared to the control group. In our study, the inoculation of CFA was effective in stimulating cell-mediated immunity. Following FHPp treatment, we observed a significant decrease in migration of inflammatory cells, in particular mononuclear cells, which suggested the possible involvement of the extract on suppression of the immune response and may be related to the inhibition of lymphocyte proliferation and nitrite production by macrophages as suggested by previous studies [2, 13].

Despite the efficacy of existing drugs for the treatment of rheumatoid arthritis, it is important to develop new immunosuppressive drugs that avoid the side effects induced by classical therapy, effects that lead to discontinuation of treatment in a high percentage of patients. Thus, in addition to demonstrating its effectiveness as an anti-inflammatory agent, the elucidation of the toxic effects of FHPp is also important. In order to observe the toxic effects after treatment, biochemical analyses were conducted with blood plasma of animals, and organs were removed and examined for their relative weight. In our study, oral treatment with FHPp caused no apparent changes in results of biochemical analyses or in organ weights, which is consistent with the observations of Pinto Coelho et al. [12] after treatment of mice for 28 days with the hydroalcoholic extract of *P. pubescens*.

We were able to demonstrate statistically significant changes in levels of glucose, cholesterol, and triglycerides. The hypoglycemic activity of the seed oil of *P. emarginatus* has been reported in a study of traditional use of plants involving 17 communities of the Alto Paraguay Bay and Guapore Valley, Mato Grosso [5]. However, it is well known that the effects of reduced levels of glucose, cholesterol and triglycerides, in the genus *Pterodon,* have not been previously reported in animal studies. 

We also observed a significant decrease in ALT levels in all groups with AIA compared to healthy animals. Although few studies have addressed this issue, this decrease can be indicative of changes in amino acid metabolism in arthritis [21]. The hepatic activity of ALT decreases up to 65.5% in Holtzman rats with AIA [22]. Ureogenesis and gluconeogenesis are able to interact in a complex way, and it is known that gluconeogenesis from the amino acids alanine and glutamine is accompanied by catabolism by nitrogen. Hence the reactions of nitrogen may be limited by the reaction of gluconeogenesis or vice versa in animals with AIA [21, 23], which explains the changes in ALT and urea levels.

An additional point is that the commonly used dose of FHPp (20 *μ*g/kg) [24] is much lower than the dose used in our study. Although several previous studies have reported the cytotoxicity of some diterpenes from plant materials [25–27], we observed no significant toxic effects, even at high FHPp doses (250 mg/kg), indicating that treatment with doses greater than those commonly used does not cause any side effects. Similar results have been demonstrated by Sabino et al. [20], who observed that the oily fraction extracted from seeds of *P. pubescens* did not induce acute toxicity in healthy rats after oral administration of doses (2, 4, and 8 g/kg) significantly higher than those ingested by humans.

The development of systemic manifestations of AIA involves the appearance of secondary polyarthritis, an increase in lymph node weight, and a decrease in body weight [28]. Because we observed an insignificant increase in the body weight of the arthritis group treated with FHPp, but observed decreased levels of glucose, cholesterol, and triglycerides, the animals were placed in metabolism cages to evaluate whether these effects were the result of metabolic changes or differences in water and food intake. We found no significant differences in food intake between the groups, leading us to believe that FHPp administration may lead to metabolic changes.

The incidences of metabolic disorders such as obesity, insulin resistance, type 2 diabetes, and dyslipidemia have increased dramatically in recent decades. Thus, research aimed at developing new drugs to contain the spread of these metabolic disorders is necessary. An investment in studies on species of the genus *Pterodon* may lead to satisfactory results.

## 5. Conclusions

The results of this study extend the findings of previous reports that show that administration of extracts and fractions obtained from species of the genus *Pterodon* exhibits anti-inflammatory activity and lacks toxicity. In addition, our study demonstrates for the first time that FHPp decreases plasma levels of glucose, cholesterol, and triglycerides, providing a rational basis for the use of this *P. pubescens *in folk medicine and encouraging the development of new drugs originating from this plant species.

## Figures and Tables

**Table 1:** FHPp effect on carrageenan-induced pleurisy.

Group	Exudate volume (mL)	% Edema inhibition	Leukocyte count (cells/mm^3^)
Control	0.80 ± 0.02	—	63400 ± 3323
FHPp 125 mg/kg	0.72 ± 0.04	10.0	59277 ± 3068
FHPp 250 mg/kg	0.57 ± 0.03^c^	28.7	51444 ± 3749
FHPp 500 mg/kg	0.59 ± 0.03^b^	26.3	48316 ± 2224^a^
Dexamethasone 0.5 mg/kg	0.20 ± 0.02^c^	75.0	17640 ± 1065^c^

Values expressed in mean ± S.E.M. (*n* = 5). ^a^ *P* < 0.05; ^b^ *P* < 0.01; ^c^ *P* < 0.001 compared to the control group. One-way ANOVA post hoc Tukey test.

**Table 2:** Effect of FHPp (250 mg/kg) on the severity of secondary injuries induced by CFA.

Day	AIA control	AIA + Tween	AIA + FHPp
11°	1.5	0.5	0.1
12°	4.2	3.5	0.4
13°	4.7	4.0	2.0
14°	4.7	4.2	3.4
15°	5.0	4.7	4.4
16°	5.0	5.0	4.7
17°	5.0	5.0	5.0

**Table 3:** Effect of FHPp (250 mg/kg) on total and differential circulating leukocytes.

Parameters	Group		
	AIA control	AIA + Tween	AIA + FHPp
Day zero			
TL	11937 ± 323	11950 ± 204	11978 ± 508
MN	9821 ± 328	9890 ± 233	9793 ± 454
PMN	2116 ± 158	2060 ± 132	2185 ± 157
21° day			
TL	51867 ± 3407	45900 ± 1088	37429 ± 2373^a^
MN	42412 ± 1940	36787 ± 370	29681 ± 1884^a^
PMN	9455 ± 1653	9113 ± 973	7748 ± 650

The data are expressed as mean ± S.E.M. (*n* = 5). TL: total leukocyte; MN: mononuclear cells; PMN: polymorphonuclear cells; the determination of the number of cells was performed on day zero (before the injection of CFA in the left hind paw) and on day 21 after induction of arthritis. ^a^ *P* < 0.01 compared to the AIA control group. One-way ANOVA post hoc Tukey test.

**Table 4:** Effect of FHPp (250 mg/kg) on biochemical measurements after 21 days of treatment.

Parameters	Groups			
	Normal	AIA control	AIA + Tween	AIA + FHPp
Glucose (mg/dL)	126.8 ± 3.5	124.7 ± 3.7	106.2 ± 5.8	101.8 ± 4.3^a,d^
Total cholesterol (mg/dL)	112.3 ± 3.1	98.0 ± 5.1	88.3 ± 9.9	64.5 ± 3.2^b,e^
Triglycerides (mg/dL)	64.6 ± 5.8	73.5 ± 4.0	51.4 ± 7.2	42.9 ± 4.8^b,c^
Creatinine (mg/dL)	0.62 ± 0.0	0.62 ± 0.0	0.61 ± 0.0	0.63 ± 0.01
Urea (mg/dL)	32.2 ± 1.4	44.0 ± 2.2	43.4 ± 5.4	45.7 ± 3.8^c^
ALT (U/L)	79.4 ± 5.7	37.0 ± 2.7^e^	31.6 ± 0.9^e^	45.3 ± 3.31^e^
AST (U/L)	102.2 ± 2.5	98.1 ± 5.3	110.4 ± 12.2	107.0 ± 2.5

Values expressed in mean ± S.E.M. (*n* = 5). ^a^ *P* < 0.01; ^b^ *P* < 0.001 compared to the AIA control group by Tukey's test; ^c^ *P* < 0.05; ^d^ *P* < 0.01; ^e^ *P* < 0.001 compared to the normal group. One-way ANOVA post hoc Tukey test.

**Table 5:** Effect of FHPp on relative organ weights (g/100 g body weight) in AIA rats.

Organ	Group		
	AIA control	AIA + Tween	AIA + FHPp
Liver	4.331 ± 0.169	3.949 ± 0.125	3.921 ± 0.185
Kidneys	0.466 ± 0.024	0.422 ± 0.008	0.412 ± 0.008
Thymus	0.070 ± 0.003	0.095 ± 0.011	0.072 ± 0.005
Spleen	0.607 ± 0.068	0.560 ± 0.052	0.493 ± 0.044
Left adrenal	0.026 ± 0.003	0.021 ± 0.002	0.022 ± 0.001
Right adrenal	0.020 ± 0.001	0.019 ± 0.002	0.021 ± 0.001
L.I.L.	0.059 ± 0.012	0.047 ± 0.002	0.038 ± 0.004
R.I.L.	0.041 ± 0.001	0.025 ± 0.004	0.036 ± 0.004
L.P.L.	0.035 ± 0.007	0.050 ± 0.005	0.038 ± 0.004
R.P.L.	0.031 ± 0.003	0.050 ± 0.011	0.033 ± 0.004

The data are expressed as mean ± S.E.M. (*n* = 5). L.I.L.: left inguinal lymph node; R.I.L.: right inguinal lymph node; L.P.L.: left popliteal lymph node; R.P.L.: right popliteal lymph node. One-way ANOVA post hoc Tukey test.
